# Reduced COVID-19 severity in Africa: a systematic review of host genetic and immunological responses to SARS-CoV-2 infection

**DOI:** 10.3389/fimmu.2026.1782808

**Published:** 2026-04-01

**Authors:** Gloria Pokuaa Manu, Joseph H. K. Bonney, Flavia Kaduni Bawa, Peter Kojo Quashie, Kwadwo Asamoah Kusi, Linda Eva Amoah

**Affiliations:** 1 West African Centre for Cell Biology of Infectious Pathogens, College of Basic and Applied Sciences, University of Ghana, Accra, Ghana; 2 Department of Biochemistry Cell and Molecular Biology, School of Biological Sciences, College of Basic and Applied Sciences, University of Ghana, Accra, Ghana; 3 Department of Immunology, Noguchi Memorial Institute for Medical Research, College of Health Sciences, University of Ghana, Legon, Ghana; 4 Department of Virology, Noguchi Memorial Institute for Medical Research, College of Health Sciences, University of Ghana, Legon, Ghana

**Keywords:** ACE2 polymorphism, African populations, COVID-19, cytokines, immune response, immunity, SARS-CoV-2 infection, T-cell responses

## Abstract

**Background:**

The emergence of severe acute respiratory syndrome coronavirus 2 (SARS-CoV-2) has had immense global consequences, leading to widespread illness, deaths, and devastated economies. Despite this, Africa has experienced a high prevalence of asymptomatic coronavirus disease 2019 (COVID-19) and mild cases. While reported cases and deaths have been lower, limited testing and undiagnosed infections make it difficult to determine the true burden of the disease. Understanding the unique immune response and the variations in genetics affect COVID-19 outcomes in African populations is important for shaping future public health responses. This review examines key immune factors and genetic variations in key host proteins that may help explain why COVID-19 was less severe in Africa.

**Methodology:**

A systematic review was conducted following PRISMA guidelines to identify studies published between 2019 and January 2026 that investigated immunological responses and genetic variations associated with COVID-19 in African populations. Literature searches were performed in PubMed, Scopus, and African Journals Online (AJOL). Inclusion criteria focused on studies reporting responses from cytokines, T-cells, antibodies or host genetic factors. After screening 4,170 records and removing duplicates, 420 studies were assessed for abstracts, and 240 full texts were reviewed. A total of 40 studies were included, and data synthesized narratively due to heterogeneity in study designs and outcomes.

**Results:**

Of the 40 studies analyzed from 19 African populations, 26 focused on immunological responses and 9 on host genetic factors. Immune studies revealed widespread pre-existing immunity, including cross-reactive antibodies (especially to the N proteins) and polyfunctional T-cell responses, likely shaped by exposure to malaria, helminths, and other coronaviruses. Severe COVID-19 cases showed elevated IL-6, TNF-α, and IFN-γ, while asymptomatic individuals had broader, milder cytokine profiles. Antibody responses were robust across disease severities, with long-lasting IgG activity. Genetic studies identified HLA-B41, B42, C16, and C17 as risk alleles, while HLA-DQB106, DQB103, and B*15 conferred protection. ACE2 polymorphisms including rs2285666, rs73635825 were reportedly prevalent in Africans and were linked to varied ACE2 expression, viral load, and disease severity.

**Conclusion:**

The findings suggest that immune and genetic adaptations in African populations may have modulated susceptibility and severity of SARS-CoV-2 infection outcomes in Africans.

**Systematic review registration:**

https://www.crd.york.ac.uk/PROSPERO/view, identifier CRD420251121731.

## 1 Introduction

The emergence of severe acute respiratory syndrome coronavirus 2 (SARS-CoV-2) and the associated coronavirus disease 2019 (COVID-19) led to a global public health crisis, with over 775 million confirmed infections and more than 7 million deaths worldwide (1). COVID-19 presents with a wide spectrum of clinical manifestations, ranging from an asymptomatic infection to severe disease that results in multi-organ damage and death (2). The disease progression follows a biphasic pattern, that begins with an early viral response phase, followed by an inflammatory phase that can result in severe complications, including acute respiratory distress syndrome (ARDS) and systemic inflammation (3).

Despite the widespread global impact of COVID-19, Africa’s experience has been different in many regions. With a population of over one billion, the continent has reported around 9.6 million COVID-19 cases. This is significantly lower than the numbers recorded in Europe (280 million), Asia (61.3 million), and the Americas (193 million). While underreporting of cases may have occurred due to limited testing and surveillance, the number of deaths reported in Africa remains considerably lower (176,000 across 55 countries) than in Europe (2.3 million), Asia (809,000), and the Americas (3 million) (1). The case-fatality ratio (CFR) in Africa remains lower than the global average (4), with a high prevalence of asymptomatic infections (5). While factors such as limited testing and surveillance may contribute to these outcomes, it is important to note that this pattern has not been uniform across the continent and countries like South Africa experienced relatively high case numbers and mortality rates. Additionally, studies comparing Africans in the diaspora to Caucasians/White Americans report of higher COVID-19 infection rates among Africans in the diaspora suggesting that environmental, unique genetic and immunological factors shape the disease outcome.

The immune response to SARS-CoV-2, though essential for viral clearance, has been implicated in disease severity. Elevated levels of pro-inflammatory cytokines such as IL-6 and TNF-α result in a cascading event referred to as cytokine storming; an excessive and uncontrolled cellular immune response where the body releases a large amount of pro-inflammatory molecules (6). This raises the question of whether the host response to SARS-CoV-2 in Africans differed from what had been observed in other global populations. Another important factor in SARS-CoV-2 infection is the interaction between the viral spike (S) protein and host ACE2 receptor. ACE2 expression is not only central to the entry of the virus but also plays a role in modulating immune response via the renin-angiotensin system (RAS). Dysregulation of the RAS is linked to increased inflammation and vascular complications (7, 8).

This review thus comprehensively summarizes available evidence on the immunological responses to SARS-CoV-2 infection and genetic variations in angiotensin converting enzyme 2 (ACE2), in African populations. We aim to identify host factors, particularly unique immune responses and genetic factors that could contribute to the less severe COVID-19 outcomes observed in Africa.

## 2 Methodology

### 2.1 Study design

A systematic review was conducted to identify studies investigating immunological responses to SARS-CoV-2 infection and genetic factors particularly ACE2 receptor properties in COVID-19 in African populations. The study followed guidelines for preferred reporting items for systematic reviews and meta-analyses (PRISMA) to ensure a rigorous and transparent methodology.

### 2.2 Search strategy

The primary databases used for the literature search were Scopus, PubMed, and Africa journals online (AJOL). The search strategy included a combination of keywords and Medical Subject Headings (MeSH) terms related to COVID-19, SARS-CoV-2 infection, immunological response and ACE2 variations (**Supplementary Table 2**). The search was focused on studies carried out on African populations and was limited to studies published between 2019 and 2024 to capture all relevant research conducted since the beginning of the COVID-19 pandemic.

### 2.3 Study selection criteria

Studies were included if they,

1. Included human participants from African populations.
2. Investigated SARS-CoV-2 infection, COVID-19 patients, or pre-pandemic samples analyzed for SARS-CoV-2 related cross-reactive immune responses.
3. Reported SARS-CoV-2 host immunological responses (e.g., antibody response, neutralization activity, cytokine profiles, T-cell responses) and/or host genetic variants (e.g., ACE2 polymorphisms, HLA alleles, susceptibility loci) across COVID-19 outcomes
4. Were primary research articles published between January 2019 and January 2026.
5. For seroprevalence studies, reported immunological characterization beyond simple seropositivity (e.g., antigen-specific antibody responses, neutralization assays, cytokine or cellular profiling).

The following studies were excluded if they,

1. Were conducted exclusively in non-African populations and does not include sub-analysis of an African cohort that meets the inclusion criteria.
2. Did not report SARS-CoV-2-specific host immunological or genetic outcomes.
3. Evaluated vaccine-induced immunity exclusively without reporting natural infection-associated responses.
4. Were reviews, commentaries, editorials, or meta-analyses.
5. Were animal or *in vitro* studies without primary human data.
6. Had inaccessible full text.
7. Had less than 10 participants sample size.

### 2.4 Screening and data extraction

#### 2.4.1 Study selection

The screening process was conducted in three stages: title screening, abstract screening, and full-text review. At each stage, two independent reviewers assessed the studies using the predefined inclusion and exclusion criteria. Any disagreements were resolved through discussion. If agreement could not be reached, a third reviewer made the final decision.

**Figure 1:**
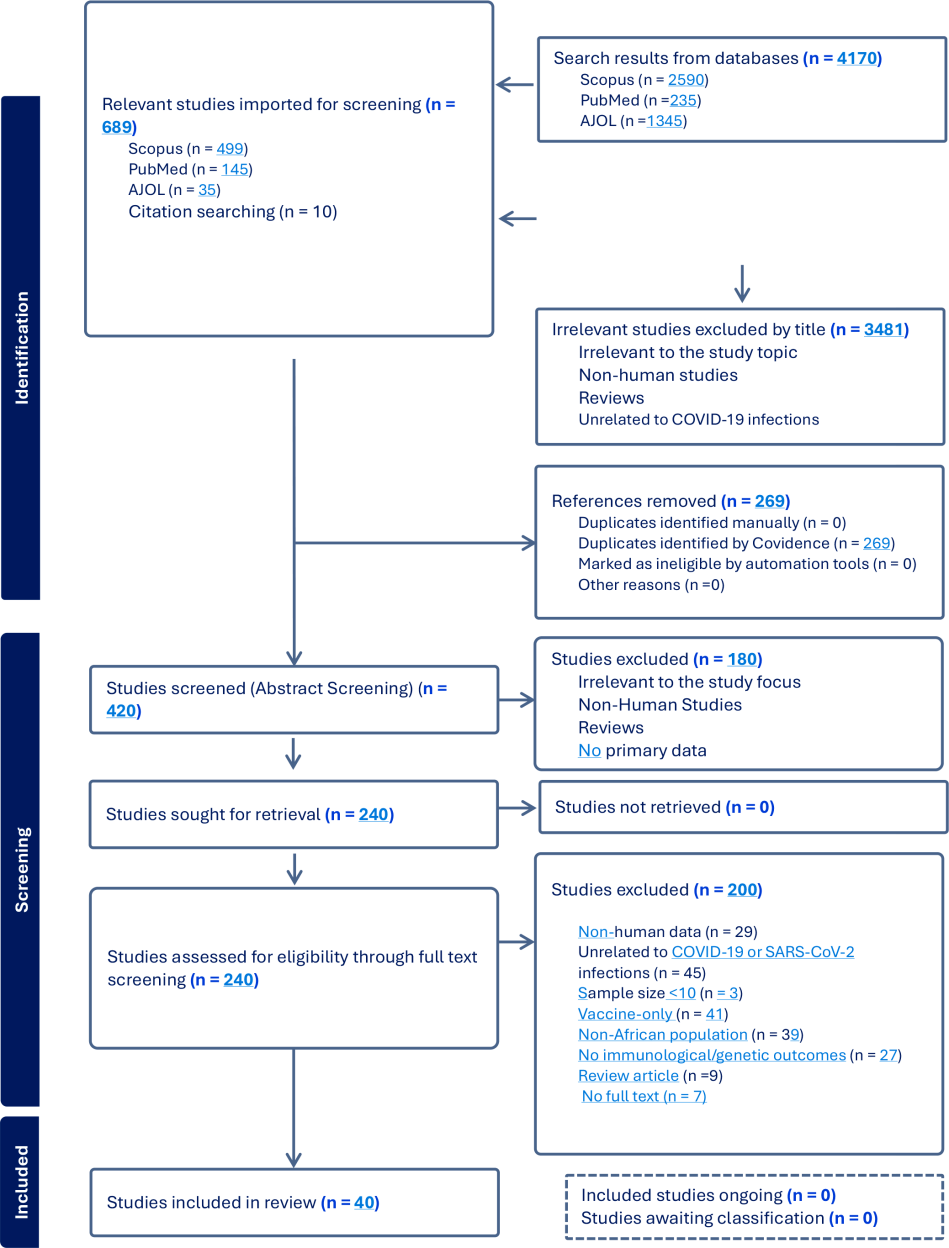
PRISMA flow diagram showing number of studies, screened, and included.

The study selection process is presented in the PRISMA flow diagram (**Figure 1**), which shows the number of records identified, screened, excluded, and included in the final analysis.

#### 2.4.2 Data extraction

Data were extracted systematically using a structured template. Two reviewers independently extracted information from each included study to ensure accuracy. Any discrepancies were resolved through discussion, consultation with a third reviewer when necessary.

The following variables were extracted:

1. Study characteristics (authors, year, country, study design, sample size)
2. Population characteristics (age, sex, and reported comorbidities)
3. Immunological outcomes (antibody isotypes and antigen specificity, neutralization activity, cytokine profiles, T-cell responses, and methods used)
4. Host genetic factors (ACE2 polymorphisms, HLA alleles)
5. Clinical phenotypes (asymptomatic, mild, moderate, severe, convalescent), where reported
6. Timing of sample collection post-infection, where available

#### 2.4.3 Quality assessment

The quality of the included studies was assessed independently by two reviewers using the Newcastle-Ottawa Scale (NOS). Any disagreements were resolved through discussion. Studies were classified as low, moderate, or high risk of bias based on predefined scoring criteria.

### 2.5 Data synthesis

The extracted data were summarized in tables to compare study characteristics, immunological findings, and host genetic factors across studies. Due to heterogeneity in study designs, laboratory methods, participant populations, outcome definitions, and timing of sample collection, a narrative synthesis was conducted, and a meta-analysis was not performed. The findings were analyzed qualitatively to identify patterns and differences in immune responses and genetic variants across African cohorts.

### 2.6 Overview of the studies from literature search

A total of 4,170 records were identified through our database searches in PubMed, Scopus, and AJOL (**Supplementary Table 2**). Following title screening, 3,481 records were excluded as irrelevant to the review focus. The remaining 689 records were imported into Covidence, a systematic review management tool for further screening.

During this process, 269 duplicate studies were detected and removed. The remaining studies (420) underwent abstract screening, after which 180 records were excluded for not meeting the inclusion criteria. A total of 240 full-text articles were assessed for eligibility. Following full-text review, 200 studies were excluded due to reasons such as non-African study populations, absence of relevant immunological or genetic outcomes. Forty (40) studies met the inclusion criteria and were included in the final review. The study selection process is summarized in the PRISMA flow diagram (**Figure 1**).

### 2.7 Risk of bias assessment

Risk of bias was assessed using the Newcastle-Ottawa Scale (NOS) (**Supplementary Table 3**). Of the 40 included studies, 17 were rated as low risk (scores 7 - 9), 23 as moderate risk (scores 4 - 6), and none of the studies were rated as high risk (scores 0 - 3). For the cross-sectional studies, a modified version of the NOS was applied to appropriately assess the risk based on sample representativeness, outcome measurement validity, control for confounders, and statistical reporting. Most studies scored well in the selection domain, but comparability was limited due to poor control for confounders (**Supplementary Table 4**).

## 3 Results

### 3.1 Characteristics of the studies included

**Figure 2:**
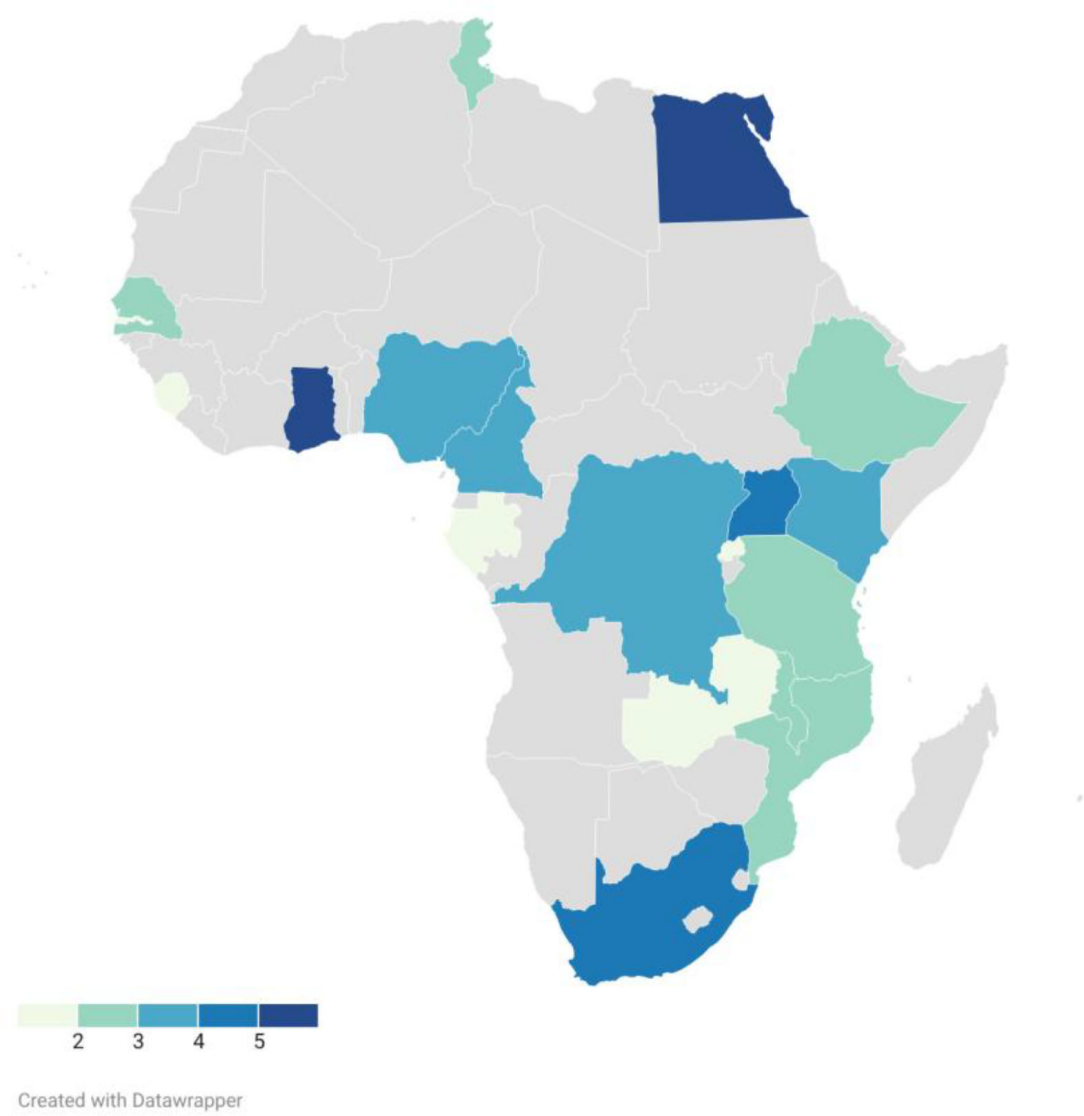
Distribution of included studies across African countries. The scale indicates the relative number of studies. The shading intensity represents the number of studies conducted in each country. Smaller numbers are indicated in light colors. Countries with no studies are identified in gray.

A total of 40 studies met the inclusion criteria following the search through 2019 to January 2026. The studies were conducted across multiple African countries, including Ghana, South Africa, Egypt, Uganda, Kenya, Malawi, Tunisia, Cameroon, Ethiopia, Nigeria, Sierra Leone, and multi-country cohorts spanning sub-Saharan Africa (**Figure 2**). Several studies included comparators from non-African populations for cross-reactivity or genetic frequency analyses. Study designs were predominantly cross-sectional and cohort-based, with a smaller number of case-control and longitudinal studies (**Table 1**). Sample were obtained from small single-center cohorts to large population-based studies exceeding 1,800 participants (**Table 1**). Sample sizes ranged from 23 to 2,504 participants.

**Table 1:** Characteristics and key findings from the included studies.

Study (author, year)	Country	Study design	Sample size (N)	Disease category	Key immunological or genetic findings
Tso et al., 2021 (10)	Tanzania, Zambia (compared with USA)	Cross-sectional	289	Pre-pandemic	Approximately 20% cross-reactivity in SSA vs ~2% USA; mainly N-directed; linked to HCoV-NL63/229E
Abdelhafiz et al., 2022 (46)	Egypt	Observational	69	Severity comparison	HLA-B*15 protective; HLA-B*41, B*42, C*16, C*17 associated with worse outcomes
Ndoricyimpaye et al., 2023 (39)	Rwanda	Prospective	217	Mild vs Severe	IFN-γ associated with severity; IL-9/IFN-γ ratio proposed as biomarker
Li et al., 2022 (14)	Africa (compared with Thailand)	Cohort (Retrospective)	2250	Pre-pandemic	Pre-pandemic S2 IgG responses; limited neutralization
Elnagdy et al., 2024 (48)	Egypt	Cross-sectional	317	Severe vs Non-severe	ACE2 rs2285666 not significant; TMPRSS2 rs12329760 protective
Borrega et al., 2021 (21)	Sierra Leone (compared with USA)	Prospective	226	Pre-pandemic	Higher cross-reactive antibodies in Sierra Leone; linked to endemic HCoVs
Serwanga et al., 2023 (28)	Uganda	Cohort (Prospective)	320	Asymptomatic vs Mild	Faster/stronger antibody response in asymptomatic; spike IgG more durable
Aguilar et al., 2024 (13)	Ghana, Mozambique	Cross-sectional (Retrospective)	602	Pre-pandemic	Higher non-specific seroreactivity in malaria-endemic settings
Gaber et al., 2024 (49)	Egypt	Cross-sectional (Prospective)	234	Severity comparison	S19P (rs73635825) genotypes linked to lower ACE2 levels and worse outcomes
Nantambi et al., 2023 (20)	Uganda	Retrospective	110	Pre-pandemic	N-directed cross-reactive responses; limited neutralization
Tufa et al., 2022 (40)	Ethiopia	Cross-sectional	260	Severe vs Mild	Elevated IL-6, IL-10, CXCL10 and inflammatory mediators in severe cases
Goda et al., 2023 (29)	Egypt	Prospective	33	Severity comparison	Severe cases had higher and more persistent IgG responses
Cherif et al., 2024 (43)	Tunisia	Cohort (Prospective)	137	Longitudinal	Anti-S-RBD IgG persisted; neutralizing antibodies in subset
Tapela et al., 2024 (30)	Ghana	Cross-sectional	291	Symptomatic vs Asymptomatic	Asymptomatic cases showed more polyfunctional T-cells
Mostafa et al., 2021 (42)	Egypt	Cross-sectional (Pilot)	40	Pediatric cases	No major Treg differences between severity groups
Bnina et al., 2023 (47)	Tunisia	Cross-sectional (Retrospective)	42	Critical illness	HLA-DQB1*06 linked to lower mortality; DQB1*03 reduced intubation
Akanmu et al., 2023 (26)	Nigeria	Cohort	134	Seroprevalence	High seroprevalence; robust N-specific T-cell responses
Fai et al., 2021 (44)	Cameroon	Cohort/Cross-sectional	1192	Seroprevalence	32% overall seroprevalence; IgM peaked at day 20, IgG at day 30; suggests substantial under-detected transmission
Samandari et al., 2023 (19)	Kenya	Cross-sectional	80	Asymptomatic	T-cell responses to ORF3a/ORF8; higher IL-10/IFN-γ ratio
Morton et al., 2021 (41)	Malawi	Cross-sectional/Prospective	87	Hospitalized	Elevated inflammatory cytokines and chemokines
Tah et al., 2023 (50)	Cameroon	Case-control	331	Susceptibility	ACE2 G8790A not significant; IL-22 genotype associated with symptoms
Adimulam et al., 2023 (12)	South Africa	Cohort	560	Ethnic comparison	ACE2 rs2285666 CC genotype associated with worse outcomes
Duah-Quashie et al., 2024 (22)	Ghana	Cross-sectional	300	Genetic diversity	ACE/ACE2 polymorphism distribution in Ghanaian population
Kato et al., 2023 (31)	Uganda	Case-control	160	Phenotype comparison	Severe cases showed elevated IFN-γ, TNF-α, IL-6, IL-10
Van Rooyen et al., 2023 (27)	South Africa	Cross-sectional	162	Prior infection vs none	T-cell proliferation to S and N common
Ugwu et al., 2024 (18)	Nigeria	Cohort/Cross-sectional	187	Vaccinated vs Convalescent	Similar binding and neutralizing responses; strong S2 T-cell responses
Tapela et al., 2022 (17)	Ghana	Cohort	144	Severity COVID-19	Cytokine differences; eotaxin higher in asymptomatic
Konlaan et al., 2022 (37)	Ghana	Cross-sectional	95	Active vs Recovered	IL-10 elevated in active infection
Pedersen et al., 2022 (11)	Gabon; Senegal	Retrospective	296	Pre-pandemic	N-specific antibodies frequent; limited neutralization
Ackah et al., 2024 (32)	Ghana	Cross-sectional	515	Severe COVID-19	No ABO association; ACE2 plasma levels varied by severity
Tso et al., 2021 (ADCC) (21)	Africa (compared with USA)	Observational	23	Functional assay	ADCC activity detected in most plasma samples
Souris 2022 (24)	Central & West Africa	Cross-sectional (Retrospective)	1655	Pre-pandemic	Pre-pandemic IgG reactivity to SARS-CoV-2 proteins
Adeniyi 2023 (78)	South Africa	Cohort (Prospective)	390	Antibody durability	Anti-N IgG persisted in subset
Namuniina 2023 (23)	Uganda	Cross-sectional (Retrospective)	29	Pre-pandemic	High frequency of cross-reactive T-cell responses
Wanjiku et al., 2026 (25)	Kenya (compared with Sweden)	Cross-sectional immunological analysis	129	Pre-pandemic samples: COVID-19 confirmed cases	Higher pre-pandemic SARS-CoV-2 spike-specific IFN-γ T-cell. Stronger S1-specific CD4+ and CD8+ T-cell responses.
de Rioja et al., 2026 (16)	Ghana; Democratic Republic of Congo; Ethiopia; Mozambique	Multi-country longitudinal cohort study	513	Asymptomatic to mild-moderate COVID-19	Robust and sustained humoral and cellular immunity for up to 12 months. longer half-lives (>50 days early decay phase) of IgG and IgA
Bhiman et al., 2025 (35)	South Africa	Population-based serological survey with neutralization assays	649	Natural SARS-CoV-2 infection	Shift in neutralizing antibody responses during transition from ancestral/D614G to Beta variant.
Jagne et al., 2025 (34)	The Gambia	Cross-sectional cohort study	349	Natural SARS-CoV-2 infection	Strong systemic neutralizing antibody responses: significant T-cell responses observed.
Müller et al., 2025 (36)	South Africa	Longitudinal study	172	SARS-CoV-2 infection (asymptomatic to critical COVID-19)	Severe COVID-19 associated with decreased ACE1 activity and increased ACE2 activity
McCormack et al., 2025 (45)	Malawi	Longitudinal population-based serosurveillance cohort	1,876	Natural SARS-CoV-2 infection	Neutralizing antibodies increased over time; hybrid immunity strongest; variant-driven shifts observed

Most studies (31) evaluated immunological outcomes, including antibody responses, cytokine profiles, and T-cell responses. A smaller subset investigated host genetic variants, primarily ACE2 and HLA polymorphisms, in relation to disease susceptibility or severity (**Table 1**). Seven (7) studies utilized samples from multiple centers (9–16). Fourteen (14) studies had pre-pandemic samples (9–11, 13, 14, 17–25) from 5,627 participants.

There were varied demographic reporting across studies. While most studies reported total sample size, age and sex distributions, they were inconsistently described, limiting direct cross-study comparability. Where reported, median ages ranged from infancy (2 months) to older adults (88 years), reflecting both pediatric and adult cohorts. Six studies did not specify age for participants (9, 11, 12, 15, 18, 20–22, 24, 26, 27). There were 4,260 (23.2%) males and 4,852 (26.4%) females participants recorded but there were no gender reports for about 50% of the total study participants. The studies captured both hospital-based severe COVID-19 cohorts and community-based asymptomatic or mild infections (9, 12, 16–18, 28–36), as well as pre-pandemic archived samples (**Supplementary Table 1**) (11, 14, 18, 19, 25, 33, 37, 38).

### 3.2 Pre-existing and cross-reactive immunity

Thirteen studies (9–11, 13, 14, 18–21, 23–25, 30) evaluated pre-pandemic samples for cross-reactive immune responses. Some multi-center studies compared the cross-reactivity of pre-pandemic sera to samples from America and Europe (9, 24, 25). Cross-reactive binding antibodies to SARS-CoV-2 antigens, particularly the N protein, were consistently detected in Africans. In several studies, about 20% of pre-pandemic samples demonstrated measurable binding reactivity to SARS-CoV-2 antigens enhanced cross-reactive cellular immunity in sub-Saharan Africa (SSA) (10, 20, 25).

Cross-reactivity was frequently attributed to prior exposure to endemic infections, including common human coronaviruses (HCoVs) (9, 14, 18, 20, 23, 24), malaria (13, 30), and helminths/protozoa (13). However, most cross-reactive antibodies lacked neutralizing capacity and were predominantly directed against the N protein. Functional neutralization against SARS-CoV-2 was generally absent in pre-pandemic sera (11, 14, 20, 21).

Limited evidence also demonstrated cross-reactive T-cell responses in individuals without confirmed prior infection, particularly against structural and accessory proteins. However, the number of studies assessing cellular cross-reactivity was small (27).

### 3.3 Immune responses during acute and convalescent COVID-19

Across studies evaluating active infection, severe COVID-19 was consistently associated with elevated pro-inflammatory cytokines, including IL-6, TNF-α, IFN-γ, and IP-10 (17, 19, 31, 37, 39–41). Reduced CCL22 levels were also reported in severe cases (40). Mild cases exhibited intermediate cytokine levels, while asymptomatic individuals generally demonstrated lower systemic inflammatory profiles (17). Anti-inflammatory cytokines such as IL-10 were also elevated in severe cases, reflecting broader immune dysregulation rather than isolated inflammatory activation. One study identified an IL-9/IFN-γ ratio associated with severity, though validation remains limited (17, 31). Additionally, chemokines like Eotaxin was higher in asymptomatic individuals and was linked to an asymptomatic phenotype alongside IL-6, IL-8, and IL-1Ra (17). A diffuse cytokine network was observed in asymptomatic cases, including IFN-α, IL-7, IL-12, and chemokines like MCP-1, MIG, and MIP-1α (16).

T-cell responses were evaluated in eight studies (17–19, 25–27, 34, 42), and demonstrated reactivity against S, N, and accessory proteins such as ORF3a and ORF8. Convalescent individuals exhibited higher T-cell proliferation compared to uninfected controls. Though the T-cell evidence remains limited, and methodologically heterogeneous, including assays and sampling timepoints, antigen pools were from the ancestral strain (17–19, 26, 27, 42).

### 3.4 Neutralizing antibodies and immune durability

Nine studies evaluated neutralizing antibody activity. Most infected individuals developed detectable neutralizing antibodies, though magnitude varied by disease severity and time since infection (10, 14, 18, 28, 29, 35, 43–45). Antibody durability varied across studies. Spike-directed IgG responses generally persisted longer than nucleocapsid antibodies, which showed more rapid decline (28, 29, 43). Differences in reported antibody magnitude between asymptomatic and severe cases likely reflect heterogeneity in sampling windows, disease stage, and assay platforms.

Recent longitudinal studies demonstrated variant-dependent differences in neutralization, with reduced activity observed against Omicron compared to ancestral strains. Hybrid immunity (infection plus vaccination) was associated with stronger and more durable neutralizing responses compared to infection alone (16, 45).

Antibody-dependent cellular cytotoxicity (ADCC) was reported in one study, indicating the presence of Fc-mediated effector functions even in individuals who lacked detectable neutralizing antibodies (10).

### 3.5 Host genetic factors

Nine studies investigated host genetic factors in Africans. Two examined HLA polymorphisms, reporting associations between specific alleles including HLA-B*41, HLA-B*42, HLA-C*16, HLA-C*17 and disease severity (46) or HLA DQB1*06, DQB1*03, HLA-B*15 and protection (46, 47). by potentially enhancing the immune response against SARS-CoV-2. However, these findings were derived from limited cohorts and specific countries.

Seven studies were found that explored ACE2 properties in COVID-19 in Africans (12, 22, 32, 36, 48–51). The rs2285666 (G8790A) variant was most frequently examined, with inconsistent associations reported across studies (12, 15, 22, 48, 50). Some cohorts demonstrated genotype associations with viral load and severity (12, 22), while others reported no significant effect on severity (48, 50).

The rs73635825 (S19P) variant was reported in African cohorts and has been associated in some studies with altered ACE2 expression or disease severity (22, 49, 51). Significant decrease in plasma ACE2 levels among moderately ill patients compared to asymptomatic individuals were also observed with this variant (32). In another study, ACE2 activity was observed to increase with severity (36).

## 4 Discussion

Corona virus disease 2019 occurred in all 55 countries in Africa however, the disease was characterized by lower numbers of severe cases and higher proportion of asymptomatic infections compared to other regions globally. As of late 2024, Africa had reported over 9 million confirmed COVID-19 cases, accounting for approximately 1.2% of the global total. The continent has also recorded around 175,000 deaths, representing about 2.5% of total global COVID-19 deaths (1, 5) and over 80% asymptomatic cases (52, 53), despite facing all the WHO’s variants of concern (54).

**Figure 3:**
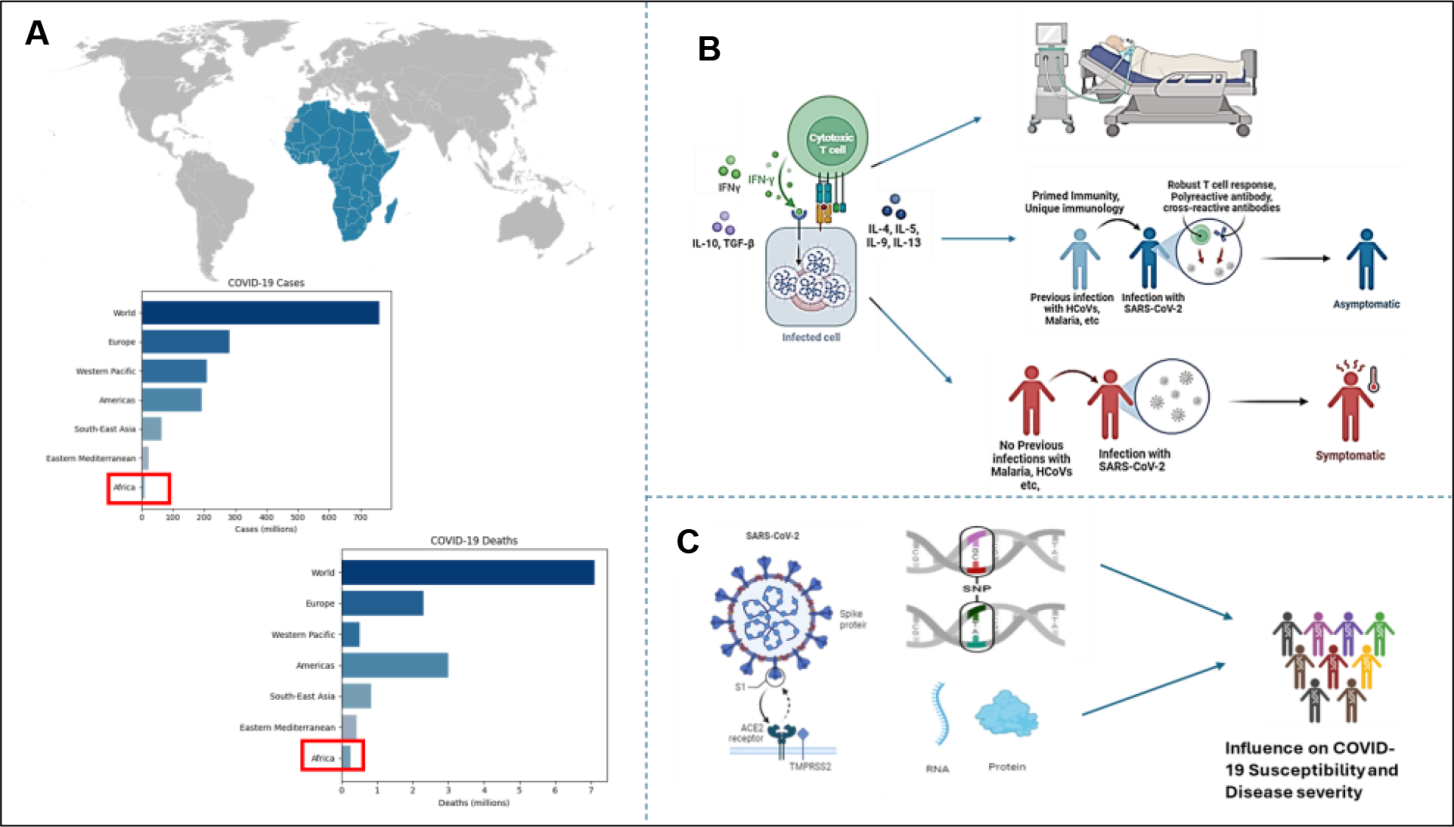
Conceptual visualization of the unique African response potentially influencing COVID-19 disease severity. **(A)** Global comparison of COVID-19 cases and deaths, highlighting the relatively lower numbers reported in Africa (1). **(B)** Proposed immunological model illustrating how prior exposure to endemic pathogens may prime immune responses, contributing to asymptomatic or mild outcomes (created in BioRender.com). **(C)** Genetic influences, including ACE2 receptor polymorphisms, may modulate susceptibility and severity of COVID-19 (created in BioRender.com).

**Figure 3** illustrates a conceptual summary of the key patterns emerging from this review. The relatively lower burden of COVID-19 cases and deaths reported in Africa compared to other global regions is highlighted. The figure also depicts how unique immune responses appear to be shaped by prior exposure to endemic infections, which may be contributing to mild disease outcomes in African. Additionally, it emphasizes the role of host genetic factors, such as ACE2 receptor polymorphisms, in influencing susceptibility to infection and disease severity. Together, these elements provide a framework for understanding the regional differences observed in COVID-19 outcomes providing a basis for understanding how specific immune and genetic factors shaped the pandemic’s impact.

### 4.1 Immunological factors modulating COVID-19 outcomes

The immunological landscape of African populations has significantly been shaped by long-term exposure to various infections, including malaria, tuberculosis, HIV and others (55). Pre-pandemic cross-reactivity was predominantly directed toward the N protein and rarely demonstrated functional neutralization (10, 11, 21, 56). Although cross-reactive and polyreactive antibodies induced by chronic exposure to parasitic infections such as Malaria (13, 57), were observed particularly in asymptomatic individuals (30, 58), the studies noted rare neutralizing activity. This suggests they may not directly prevent infection but could contribute to immune modulation. However, while prior exposure to endemic infections (9, 59) may contribute to immune priming, current evidence does not establish a direct protective effect against severe COVID-19.

Recently, polyreactive antibodies have been shown to exhibit broad antibacterial activity by binding to diverse bacterial species, enhancing immune defense mechanisms such as complement activation and phagocytosis (60). This ability of polyreactive antibodies to recognize structurally unrelated pathogens suggests a vital role in shaping early immune responses to infections. Their regulatory effect may also limit excessive inflammation contributing to immune modulation (58, 61). Hence the chronic exposure to a high burden of parasitic infections appears to be a significant factor contributing to a broader, less specific antibody repertoire that can cross-react with SARS-CoV-2 antigens but direct links to COVID-19 severity require further investigation.

Beyond traditional neutralization, non-neutralizing antibodies contribute significantly to viral control, particularly in populations with a broader immune response repertoire (62). The presence of ADCC, even in the absence of neutralizing antibodies confirms this compensatory mechanism (63). Both neutralizing and non-neutralizing antibodies have been reported to contribute to viral control through the induction of Fc-mediated effector functions against SARS-CoV-2 (64–66). While some of the studies shows overlap, it also illustrates how ADCC and other effector functions can be present and contribute to viral control even when neutralizing antibody titers are low or undetectable (64, 66). However, the extent to which these mechanisms influence clinical outcomes remains uncertain and requires functional validation as well as more studies in this area.

Severe disease was consistently associated with elevated inflammatory cytokines, similar to what has been reported globally (67, 68). Elevation of markers such IL-6, IL-10, IFN-γ and TNF-α in severe COVID-19 suggests a common immune dysregulation that drives severe COVID-19 across different populations (17, 39, 67, 68). Whiles higher IL-9/IFN-γ ratio was found to be a predictive biomarker for severe disease (35). This shows the importance of early immune modulation in managing COVID-19. Eotaxin, a chemokine involved in the regulation of eosinophils, particularly in allergic and inflammatory reactions (69) has been associated with parasitic infections with elevated levels of eotaxin reported in individuals exposed to hookworm infestation (70), malaria (71) and filariasis (72), reflecting an eosinophil-driven immune response typical of chronic parasitic exposure. The observation of elevated eotaxin levels in some asymptomatic individuals is noteworthy as this may reflects prior parasitic exposure, host baseline immune state, or a direct role in SARS-CoV-2 infection though remains unclear and studies are limited.

Evidence for SARS-CoV-2-specific T-cell responses in African populations remains limited and methodologically diverse. T-cell responses directed against accessory proteins such as ORF3a and ORF8 were reported in some African cohorts, particularly among asymptomatic individuals. These findings indicate possible broader antigen recognition pattern; however, the study did not directly assess cytotoxic function or viral clearance. Hence, while such responses are immunologically interesting, their protective significance cannot be definitively established. Further research using functional assays would be needed to confirm the protective or cytotoxic role of these responses.

The decline of nucleocapsid-specific antibodies observed in African cohorts is consistent with findings from other regions, where anti-nucleocapsid IgG levels have also been shown to decrease within months after infection (73–75). This broader evidence indicates that waning of N-protein antibodies is a widely reported feature of SARS-CoV-2 immunity rather than a phenomenon unique to African populations. Neutralizing antibody responses were influenced by viral variant and exposure history (16, 34, 35, 45). Reduced neutralization against Omicron compared to ancestral strains was observed, consistent with global findings (76, 77). Although strong responses to accessory proteins (23), long-term antibody persistence (78) to structural proteins, and high prevalence of pre-existing serological cross-reactivity to SARS-CoV-2 antigens (79, 80) were observed, their direct contribution to viral clearance requires further functional validation.

### 4.2 Host genetic factors modulating COVID-19 outcomes

Host genetic associations were identified in limited cohorts, primarily focusing on HLA and ACE2 polymorphisms. Genetic variation in HLA and ACE2 has been explored as a possible contributor to inter-individual differences in COVID-19 severity. While some alleles (HLA-B*15 and HLA-B*41/HLA-B*42) were associated with disease protection or protection (81), evidence remains limited and geographically concentrated. Additionally, allele frequency varies widely between and within populations, and most findings are derived from relatively small cohort studies. Larger, multi-country genomic studies are required to validate these associations and clarify their clinical relevance.

The risk and protective variants of ACE2 observed in Africans provide a genetic framework that may also explains the disparities in COVID-19 outcomes. According to recent research, ACE2 polymorphisms can influence the expression (82) and function of the ACE2 protein (83), receptor affinity for viral binding (83), viral load (12) and downstream immune responses in SARS-CoV-2 infection (51, 84). ACE2 polymorphisms, including rs73635825 (S19P) and rs2285666, have been investigated for potential effects on receptor expression and spike binding affinity (12, 15, 51, 85). While some studies suggest altered ACE2 expression or receptor binding associated with these variants, potentially enhancing viral entry and worsening infection (22, 49, 51, 82), reported associations with disease severity are inconsistent and studies limited.

## 5 Conclusion

**Figure 4:**
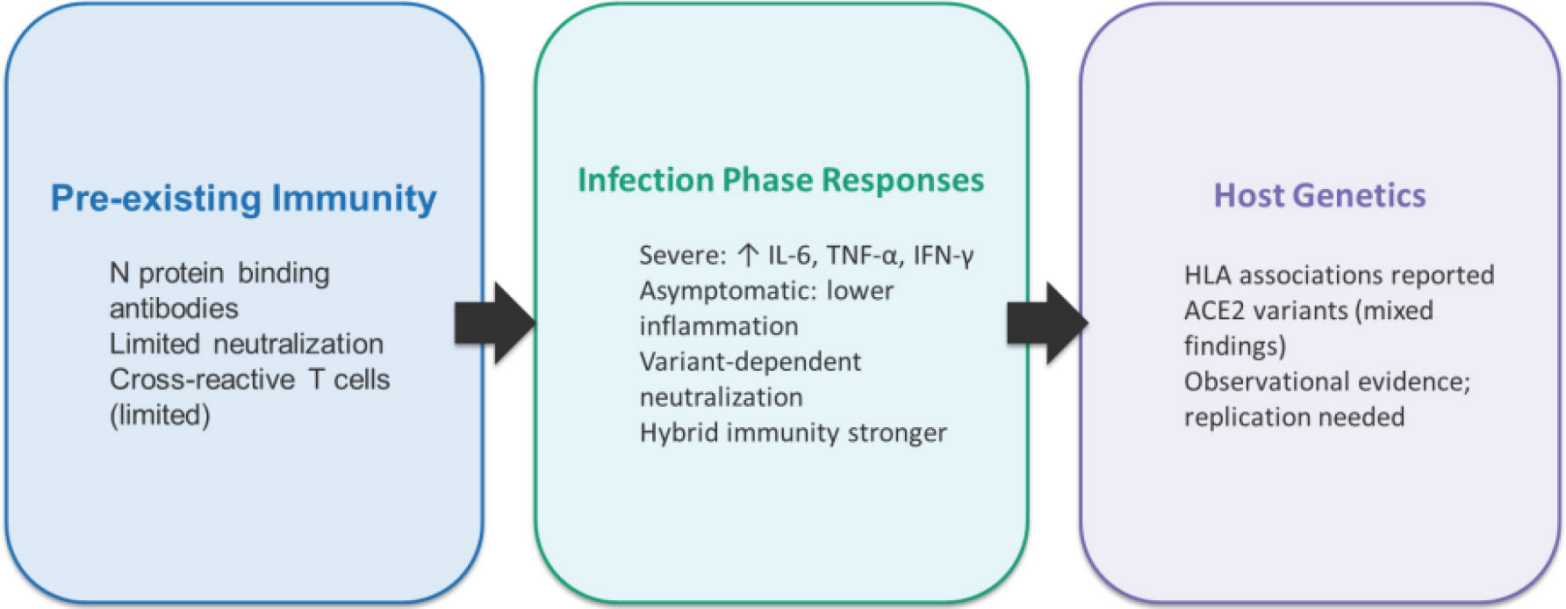
Overview of immunological and host genetic evidence from African SARS-CoV-2 studies (2019–2026). Evidence from African cohorts demonstrates pre-existing cross-reactive binding antibodies with limited neutralizing capacity, inflammatory cytokine signatures associated with severe disease, and preliminary host genetic associations involving HLA and ACE2 variants.

This review examined existing studies to identify unique immunological and genetic factors in African populations that may have contributed to the lower severity of COVID-19 in the region. Our findings shows that African cohorts exhibit detectable pre-existing cross-reactive immunity, inflammatory patterns associated with COVID-19 severity, and variant-dependent neutralizing responses (**Figure 4**). Pre-existing cross-reactive immunity shaped by past exposure to other infections were more targeted at the N protein than the S protein, and in some cases accessory proteins. Antibodies from these past exposures rarely neutralized SARS-CoV-2 in Africans. This suggests that while pre-existing immunity was present, it may not significantly explain protection against severe infection. Host genetic associations involving HLA and ACE2 variants with SARS-CoV-2 infection outcomes have been reported but studies remain limited and require replication in larger, multi-country studies.

This review observes limitations in methodology across studies, including heterogeneous sampling windows, inconsistent demographic reporting, small single-center cohorts, and variability in assay platforms. Limited reporting of age and sex also restricts cross-study comparability. Future research should prioritize large, longitudinal, multi-country African cohorts using standardized immunological and genomic methods to validate these associations. Such studies are essential to clarify whether the identified host factors meaningfully influence disease severity, vaccine responses, and long-term immunity.

## Data Availability

The original contributions presented in the study are included in the article/**Supplementary Material**. Further inquiries can be directed to the corresponding authors.
