# Supplementary material for: Reduced COVID-19 severity in Africa: a systematic review of host genetic and immunological responses to SARS-CoV-2 infection
Source: Front Immunol. 2026 Apr 1;17:1782808. doi: 10.3389/fimmu.2026.1782808 (PMC13079709; doi:10.3389/fimmu.2026.1782808)
Supplement: Supplementary file 1 [file Table1.docx]

***Supplementary Materials***

# **Supplementary Data**

Table S1: Summary of the characteristics of the studies included

# **Supplementary Tables and Figures**

## **Supplementary Tables**

Table S2: Search strategies for literature search

| Search term/Keywords | Database | Number of publications | Relevant studies exported to Covidence for screening |  |
| --- | --- | --- | --- | --- |
| Search strategy 1 | PubMed | 11 | 11 |  |
|  | Scopus | 673 | 152 |  |
|  | AJOL | 296 | 6 |  |
| Search strategy 2 | PubMed | 14 | 14 |  |
|  | Scopus | 790 | 120 |  |
|  | AJOL | 584 | 5 |  |
| Search strategy 3 | PubMed | 9 | 9 |  |
|  | Scopus | 481 | 142 |  |
|  | AJOL | 236 | 8 |  |
| Search strategy 4 | PubMed | 199 | 109 |  |
|  | Scopus | 441 | 41 |  |
|  | AJOL | 133 | 7 |  |
| Search strategy 5 | PubMed | 2 | 2 |  |
|  | Scopus | 205 | 44 |  |
|  | AJOL | 96 | 9 |  |
| TOTAL |  | 4,170 | 679 |  |
| **Search Strategy 1** | | | | |
| ("COVID-19" OR "SARS-CoV-2") AND ("immunological factors" OR "immune response" OR "ACE2 ") AND ("Africa" OR "African populations" OR "Algeria" OR "Angola" OR "Benin" OR "Botswana" OR "Burkina Faso" OR "Burundi" OR "Cabo Verde" OR "Cameroon" OR "Central African Republic" OR "Chad" OR "Comoros" OR "Congo" OR "Democratic Republic of the Congo" OR "Djibouti" OR "Egypt" OR "Equatorial Guinea" OR "Eritrea" OR "Eswatini" OR "Ethiopia" OR "Gabon" OR "Gambia" OR "Ghana" OR "Guinea" OR "Guinea-Bissau" OR "Ivory Coast" OR "Kenya" OR "Lesotho" OR "Liberia" OR "Libya" OR "Madagascar" OR "Malawi" OR "Mali" OR "Mauritania" OR "Mauritius" OR "Morocco" OR "Mozambique" OR "Namibia" OR "Niger" OR "Nigeria" OR "Rwanda" OR "Sao Tome and Principe" OR "Senegal" OR "Seychelles" OR "Sierra Leone" OR "Somalia" OR "South Africa" OR "South Sudan" OR "Sudan" OR "Tanzania" OR "Togo" OR "Tunisia" OR "Uganda" OR "Zambia" OR "Zimbabwe") | | | | |
| **Search Strategy 2** | | | | |
| ("COVID-19" OR "SARS-CoV-2") AND ("immunological response" OR "immune response" OR “immunity" OR “ACE2" OR “ACE2 polymorphism” OR “ACE2 variants”) AND ("Africa" OR "African populations") | | | | |
| **Search Strategy 3** | | | | |
| ("Africa" OR "African populations") AND ("immune response" OR "cytokines" OR "T-cells" OR "immune diversity" OR "ACE2 receptor" OR "angiotensin-converting enzyme 2" OR "SARS-CoV-2 receptor") AND “COVID-19” | | | | |
| **Search Strategy 4** | | | | |
| “Immune responses” OR “Immunological Responses” OR “Immunogenic factors” AND “COVID-19” OR “SARS-CoV-2” AND “Africa” OR “Africans” OR “African population” | | | | |
| **Search Strategy 5** | | | | |
| (“ACE2” OR "ACE2 variants" OR "ACE2 Polymorphisms" OR "ACE2 SNPS") AND ("COVID-19" OR "SARS-CoV-2") AND (Africa OR Africans OR "African population") | | | | |

Table S3: Risk of Bias Assessment Questionnaire and Score

| Item No. | Domain | Assessment Question | Score (★ / 0) |
| --- | --- | --- | --- |
| 1 | Selection | Is the case definition adequate? |  |
| 2 | Selection | Are the cases representative? |  |
| 3 | Selection | Is the selection of controls appropriate? |  |
| 4 | Selection | Is the definition of controls adequate? |  |
| 5 | Comparability | Did the study control for the most important confounding factor (e.g. age, sex)? |  |
| 6 | Comparability | Did the study control for additional confounders? |  |
| 7 | Exposure | Was the ascertainment of exposure (e.g. ACE2 genotype or immune marker) valid? |  |
| 8 | Exposure | Was the same method used to ascertain exposure in cases and controls? |  |
| 9 | Exposure | Was the non-response rate the same in both groups, or acceptably low? |  |

**Table S4: Risk assessments of Bias for the Studies Included**

| **Study (Author, Year)** | **Selection (max 4★)** | **Comparability (max 2★)** | **Exposure/Outcome/ Missing data/ Statistical Analysis (max 3★)** | **Total Score (max 9★)** | **Risk Level** |
| --- | --- | --- | --- | --- | --- |
| Tso et al., 2020 | **★★★** | **★** | **★★** | **6** | Moderate Risk |
| Abdelhafiz et al., 2022 | **★★★★** | **★** | **★★** | **7** | Low Risk |
| Ndoricyimpaye et al., 2023 | **★★★** | **★** | **★★** | **6** | Moderate Risk |
| Li et al., 2022 | **★★★** |  | **★★★** | **6** | Moderate Risk |
| Elnagdy et al., 2024 | **★★★★** | **★★** | **★★** | **8** | Low Risk |
| Borrega et al., 2021 | **★★★** | **★** | **★★** | **6** | Moderate Risk |
| Serwanga et al., 2023 | **★★★** | **★** | **★★★** | **7** | Low Risk |
| Aguilar et al., 2024 | **★★★** | **★** | **★★★** | **7** | Low Risk |
| Gaber et al., 2024 | **★★★★** | **★** | **★★** | **7** | Low Risk |
| Nantambi et al., 2023 | **★★★** | **★** | **★★** | **6** | Moderate Risk |
| Tufa et al., 2022 | **★★★** |  | **★★★** | **6** | Moderate Risk |
| Goda et al., 2023 | **★★** |  | **★★★★** | **6** | Moderate Risk |
| Cherif et al., 2024 | **★★★** |  | **★★★★** | **7** | Low Risk |
| Tapela et al., 2024 | **★★★★** | **★** | **★★** | **7** | Low Risk |
| Mostafa et al., 2021 | **★★★** | **★** | **★★** | **6** | Moderate Risk |
| Bnina et al., 2023 | **★★** | **★** | **★★★** | **6** | Moderate Risk |
| Akanmu et al., 2023 | **★★★** | **★** | **★★★** | **7** | Low Risk |
| Fai et al., 2021 | **★★★** |  | **★★** | **5** | Moderate Risk |
| Samandari et al., 2023 | **★★★** | **★** | **★★★** | **7** | Low Risk |
| Morton et al., 2021 | **★★★★** | **★★** | **★★** | **8** | Low Risk |
| Tah et al., 2023 | **★★★** | **★** | **★★** | **6** | Moderate Risk |
| Adimulam et al., 2023 | **★★** | **★** | **★★★** | **6** | Moderate Risk |
| Duah-Quashie et al., 2024 | **★★** | **★** | **★★★** | **6** | Moderate Risk |
| Kato et al., 2023 | **★★★** | **★** | **★★** | **6** | Moderate Risk |
| Van Rooyen et al., 2023 | **★★★** | **★** | **★★** | **6** | Moderate risk |
| Ugwu et al., 2024 | **★★** | **★** | **★★** | **5** | Moderate Risk |
| Tapela et al., 2022 | **★★★** | **★** | **★★** | **6** | Moderate Risk |
| Konlaan et al., 2022 | **★★★** | **★** | **★★★** | **7** | Low Risk |
| Pedersen et al., 2022 | **★★★★** | **★** | **★★★** | **8** | Low Risk |
| Ackah et al., 2024 | **★★★** | **★** | **★★** | **6** | Moderate Risk |
| Tso et al., 2021 | **★★★** | **★** | **★★** | **6** | Moderate Risk |
| Souris 2022 | **★★★** | **★** | **★★** | **6** | Moderate Risk |
| Adeniyi 2023 | **★★★** | **★★** | **★★** | **7** | Low Risk |
| Namuniina 2023 | **★★** | **★★** | **★★** | **6** | Moderate Risk |
| Wanjiku et al., 2026 | **★★** | **★** | **★★★** | **6** | Moderate |
| de Rioja et al., 2026 | **★★★★** | **★★** | **★★★** | **9** | Low |
| Bhiman et al., 2025 | **★★★** | **★** | **★★** | **6** | Moderate |
| Jagne et al., 2025 | **★★★★** | **★** | **★★★** | **8** | Low |
| Müller et al., 2025 | **★★★** | **★★** | **★★★** | **8** | Low |
| McCormack et al., 2025 | **★★★★** | **★** | **★★★** | **8** | Low |

Risk of bias assessment of included studies. Scores 7-9 considered as low risk, scores 4-6 moderate risk, and scores 0-3 considered as high risk.
